# Case Report: Application of bronchoalveolar lavage fluid morphology in the diagnosis of pulmonary fat embolism

**DOI:** 10.3389/fmed.2026.1818286

**Published:** 2026-04-13

**Authors:** Xiang Li, Yanzi Pan, Xiaojuan Qian, Hua Wang, Yuli Zhou

**Affiliations:** 1 Department of Laboratory Medicine, Affiliated Sixian People's Hospital, Suzhou, Anhui, China; 2 School of Medical Technology and Information Engineering, Zhejiang Chinese Medical University, Hangzhou, China; 3 Department of Laboratory Medicine, Affiliated Hangzhou First People's Hospital, School of Medicine, Westlake University, Hangzhou, China; 4 Department of Laboratory Medicine, Affiliated the Second People's Hospital of Qinzhou, Qinzhou, China

**Keywords:** aesthetic surgery complications, bronchoalveolar lavage fluid morphology, liposuction surgery, pulmonary fat embolism, Sudan III staining

## Abstract

**Background:**

Pulmonary fat embolism (PFE) is a serious complication of liposuction surgery, characterized by atypical clinical presentation and considerable diagnostic difficulty. Morphological examination of Bronchoalveolar Lavage Fluid (BALF) can provide critical evidence for diagnosis. This article reports a rare delayed-onset case, highlighting the diagnostic value of this examination and the importance of risk prevention and management in plastic surgery.

**Case presentation:**

A 25-year-old female was admitted with a 4-day history of chest tightness, which worsened after eating and at night. The patient had undergone liposuction surgery half a month prior. Laboratory findings revealed elevated D-dimer levels and decreased total protein and albumin. Chest Computed Tomography (CT) suggested chronic inflammation with fibroproliferative changes in the left lower lobe. Bronchiectasis was initially diagnosed; however, anti-infective therapy was ineffective. Microscopic examination of BALF revealed fat droplets and macrophages phagocytosing fat particles, with positive Sudan III staining. In conjunction with contrast-enhanced pulmonary CTA, the patient was ultimately diagnosed with pulmonary fat embolism secondary to liposuction. After receiving symptomatic and supportive treatment, the patient's symptoms resolved, and she was discharged.

**Conclusion:**

This case indicates that pulmonary fat embolism following liposuction is prone to being misdiagnosed. BALF morphological examination can effectively assist in early diagnosis and gain valuable time for treatment. Meanwhile, this case also reminds us of the significance of risk assessment in aesthetic and plastic surgery. Clinicians should remain vigilant regarding atypical postoperative symptoms to prevent missed diagnoses and misdiagnoses.

## Background

Fat embolism refers to partial or complete vascular obstruction in multiple organs due to the presence of lipid droplets. When fat globules are present in the pulmonary circulation, it is termed pulmonary fat embolism (PFE). This condition most commonly occurs following severe trauma, especially long bone fractures and soft tissue crush injuries involving adipose tissue, such as blunt trauma, liposuction, and fat grafting. In addition, certain diseases (such as sickle cell disease and Duchenne muscular dystrophy) and parvovirus infections may also trigger fat embolism. Clinically, death resulting from fat embolism is not uncommon, as it may occur as a complication of minor trauma, surgical procedures, or other treatments; therefore, it warrants significant clinical attention. However, when pulmonary fat embolism occurs, the clinical manifestations are often atypical, posing challenges to diagnosis and treatment. Classic PFE occurs within 24–48 h after injury, but atypical and delayed cases are highly prone to missed diagnosis. In fact, persistent abnormalities on electrocardiography, echocardiography, and chest CT strongly suggest the possibility of pulmonary embolism (1). Although these findings cannot serve as diagnostic criteria for pulmonary embolism, they are helpful in differentiating other cardiopulmonary diseases that present with similar symptoms. BALF examination can directly identify characteristic fat droplets and associated cellular morphological changes, confirm the presence of lipid components, and indicate acute inflammation and injury responses in lung tissue. It provides direct morphological evidence and serves as an important adjunct for clinical diagnosis. This technique plays an irreplaceable role in early diagnosis and differential diagnosis, especially in patients with inconspicuous symptoms and unremarkable laboratory findings. If a hospital suspects pulmonary fat embolism, further pulmonary angiography may be performed, which could help save the patient's life (2). This case report describes a delayed-onset pulmonary fat embolism following liposuction. The case illustrates the diagnostic challenges of pulmonary fat embolism, highlights the critical role of morphological examination of BALF in the diagnostic process, and emphasizes the importance of risk assessment and prevention in cosmetic surgery.

## Case presentation

**Figure 1:**
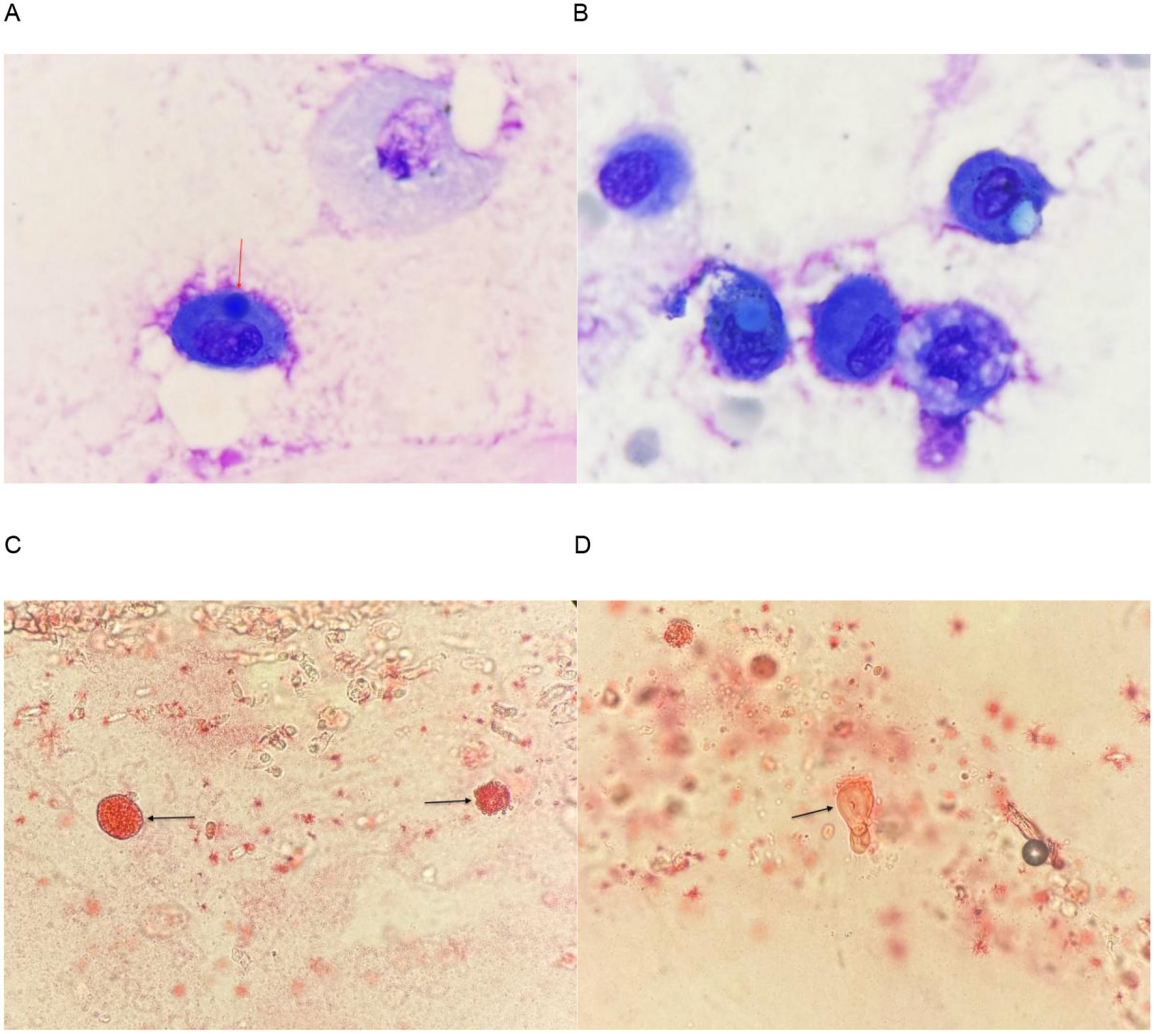
**(A, B)** BALF, Wright-Giemsa Stain 1,000×. Alveolar phagocytes phagocytizing spherical substances are visible, which are unstained, light blue-stained, or dark blue-stained. **(C, D)** BALF, Sudan III Stain 400×. **(C)** Nucleated cells are scattered, and the cytoplasm of alveolar macrophages is filled with lipid substances showing a foamy appearance, which is orange-red after Sudan III staining. **(D)** Lipid droplets of varying sizes or irregular shapes are visible, positive for Sudan III.

The patient is a 25-year-old female who was admitted with a 4-day history of chest tightness without obvious predisposing factors. The symptoms worsened after meals and at night and were not associated with physical activity. Physical examination revealed no significant abnormalities. She had a history of bronchopneumonia and pulmonary tuberculosis more than 10 years prior. After admission, relevant laboratory examinations were performed. Routine blood tests showed a white blood cell count of 8.3 × 10^9^/L with an elevated neutrophil ratio (68.1%). D-dimer level was 991 μg/L (↑). Biochemical tests revealed only two abnormal parameters: total protein 59.7 g/L (↓) and albumin 35.1 g/L (↓). Quantitative troponin, B-type natriuretic peptide, liver and kidney function, serum lipase and amylase, coagulation function, erythrocyte sedimentation rate, immunoglobulin levels, and lymphocyte subsets were all within normal reference ranges. Ultrasound examinations of the urinary system and the upper abdomen (including the liver, gallbladder, pancreas, and spleen) showed no significant abnormalities. Electrocardiogram revealed sinus arrhythmia, anteroseptal ST-T changes, and anterolateral T-wave changes. Chest CT indicated chronic inflammation with fibroproliferative changes in the left lower lobe, along with localized mild bronchiectasis. Pulmonary function testing suggested mild restrictive ventilatory dysfunction. The initial diagnosis was bronchiectasis. After treatment with cephalosporin antibiotics, the symptoms did not improve. Secondary care was provided, along with symptomatic treatment including nebulization and oxygen therapy. On the fourth day of hospitalization, further BALF examination was performed: cryptococcal antigen test was negative; tests for Mycobacterium tuberculosis and rifampicin resistance gene, Mycobacterium tuberculosis DNA (Xpert), GM test, acid-fast bacillus smear of bronchoscopic brushings, and multiplex combined testing for 153 respiratory pathogens in BALF were all negative. Morphological analysis of BALF showed: a nucleated cell count of 428/μl, including 60% neutrophils, 3% lymphocytes, 34% alveolar macrophages, and 3% eosinophils. Fat droplets were readily observable in the lavage fluid, and macrophages phagocytizing fat granules were detected. After Sudan III staining, they appeared orange–red (specific staining for neutral fat), confirming the presence of lipid droplets in the lungs (Figure 1). Combined with the morphological characteristics of BALF (especially the phagocytosis of small fat droplets by macrophages), pulmonary fat embolism was highly suspected. This finding provided key morphological evidence for the diagnosis. Enhanced pulmonary vascular CTA was completed, suggesting possible pulmonary embolism in the left lower lobe pulmonary artery, further confirming the diagnosis of pulmonary fat embolism.

**Figure 2:**
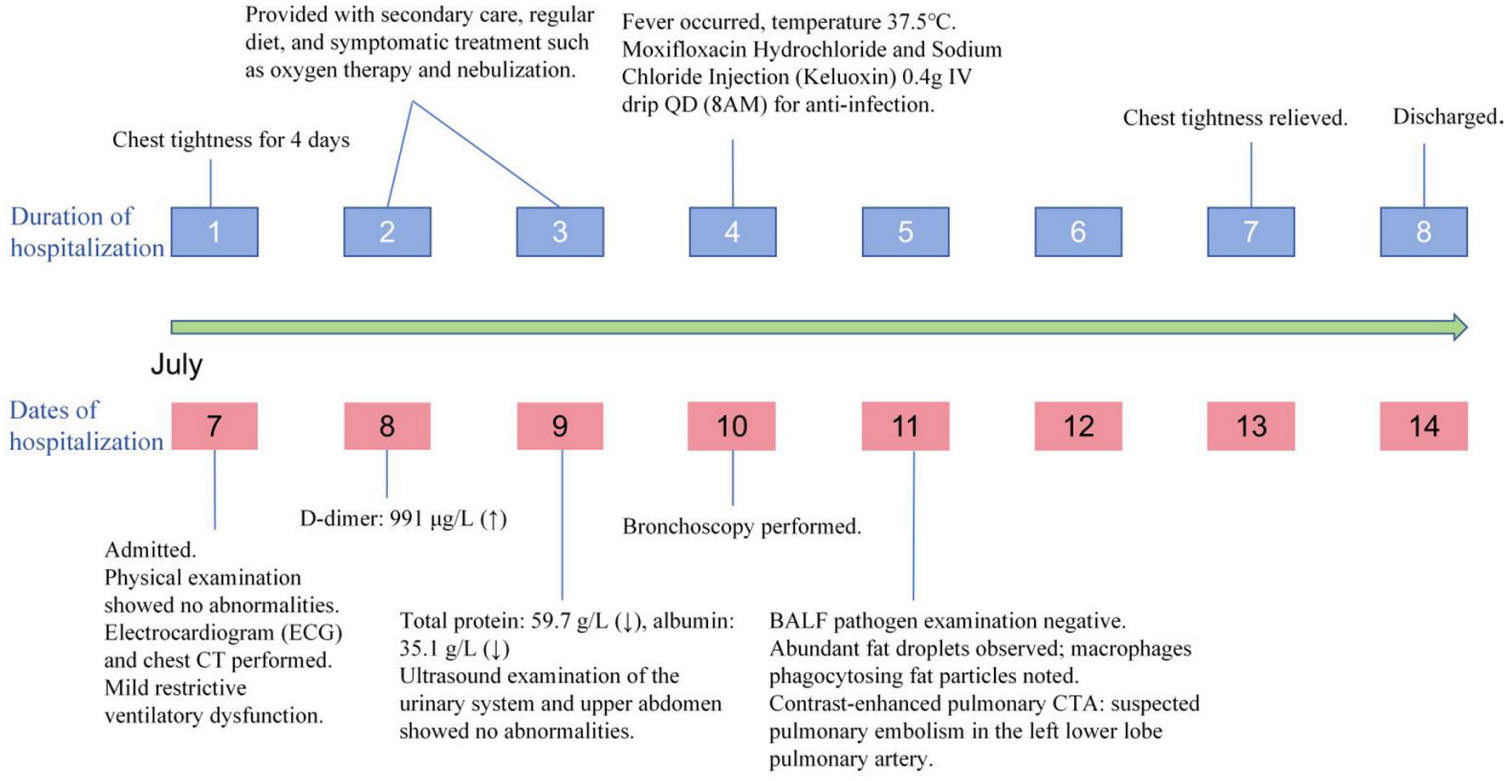
Timeline of clinical course.

Considering the surgical history, the patient had undergone liposuction surgery half a month prior. Between the surgery and the acute onset of this illness, she experienced fever, followed by gradually worsening chest tightness and occasional dizziness. No other trauma, surgery, infection, or acute illness was reported. After differential diagnosis to exclude conditions such as fat embolism syndrome, bacterial pneumonia, mycoplasma pneumonia, bronchial asthma, and cardiac insufficiency, the findings from bronchoalveolar lavage fluid cytomorphology, chest CT, and pulmonary angiography collectively supported the diagnosis of pulmonary fat embolism. Following symptomatic treatment, the patient's chest tightness was alleviated compared with before, and she was discharged. Two weeks later, the patient returned for follow-up to assess recovery; she achieved clinical cure after treatment with no long-term complications. The patient later sought care at another hospital, and further rehabilitation data were not available. The timeline is shown in Figure 2.

## Discussion and conclusions

With the improvement of living standards, people's pursuit of body contour has been on the rise, and liposuction surgery has gradually gained popularity, leading to a surge in the demand for surgical intervention. However, pulmonary fat embolism has become one of the most severe complications of liposuction and fat grafting. During the operation, damage to adipose tissue and perforation of small blood vessels can generate lipid fragments, which may cause lung injury after entering the venous system (1). The severity of the ventilation–perfusion imbalance caused by it is proportional to the circulating fat load. It typically presents with signs and symptoms such as dyspnea, chest pain on inspiration, palpitations, decreased blood oxygen saturation, cyanosis, tachypnea, and tachycardia (3).

In clinical practice, the diagnostic process for pulmonary fat embolism is highly challenging due to the low specificity and sensitivity of symptoms, relatively low prevalence, and the lack of definitive diagnostic modalities and standardized diagnostic criteria. Diagnosis is often made by exclusion, typically based on clinical manifestations combined with imaging and laboratory findings. Up to 5% of fat embolism cases are caused by non-traumatic conditions, such as hemoglobinopathies (such as sickle cell disease), severe acute pancreatitis, diabetes mellitus, osteomyelitis, and long-term corticosteroid therapy. Therefore, a thorough medical history, physical examination, and comprehensive laboratory workup are essential upon patient admission. The symptoms of pulmonary fat embolism closely resemble those of numerous surgery-related complications, including adult respiratory distress syndrome, pulmonary edema, aspiration pneumonia, drug reactions, and transfusion-related acute lung injury. Consequently, prompt differentiation of pulmonary fat embolism from these conditions is particularly difficult. Regarding FES, its clinical manifestations typically appear 24–48 h after the initial insult. The widely used Gurd and Wilson criteria for diagnosing FES are based on the classic triad: hypoxemia/hypoxia (present in 95% of cases), central nervous system depression (60% of cases), and petechial hemorrhagic rash (33% of cases). However, only 3%−4% of patients present with the complete clinical triad (4). Diagnosis of FES requires two major criteria, or one major criterion with four minor criteria (5). In the present case, the patient did not exhibit a petechial rash, remained mentally alert, developed a low-grade fever (37.5 °C) on the third day of hospitalization, and showed no significant ophthalmic abnormalities. Multiple findings did not meet the diagnostic criteria, thus allowing FES to be ruled out. Moreover, treatment strategies differ significantly among these conditions, further adding to the diagnostic complexity. Additionally, patients often present with acute respiratory distress or hemodynamic instability, leading them to be admitted to emergency departments rather than the facility where the liposuction was performed. In such situations, the lack of reliable surgical history further exacerbates the diagnostic difficulty.

In laboratory examinations, decreased oxygen saturation, leukocytosis, anemia, thrombocytopenia, and elevated D-dimer levels are common abnormalities. Chest CT and cytomorphological analysis of BALF are of great diagnostic value. Bronchoalveolar lavage is both a diagnostic and therapeutic procedure that facilitates the removal of metabolic products, inflammatory necrotic material, and other debris from the deep lung, thereby achieving therapeutic effects. Moreover, BALF is derived from the deep lung and reflects the true condition of the disease, making it a valuable specimen. In the present case, the patient was admitted with a 4-day history of chest tightness. Examinations revealed mild restrictive ventilatory dysfunction, with no typical signs or symptoms. The initial diagnosis was bronchiectasis, and other laboratory findings were unremarkable, making it difficult to establish a definitive diagnosis and treatment direction. Additionally, the patient did not present within the typical onset window following the invasive procedure, further confounding the clinical diagnosis. It was not until fat droplets were identified in the patient's BALF, combined with the history of liposuction performed half a month earlier, that a diagnosis of mild pulmonary fat embolism secondary to liposuction was suspected. After differential diagnosis to exclude other suspected conditions and in conjunction with chest CT findings of mild inflammation and fibrosis, as well as contrast-enhanced pulmonary CTA showing suspected pulmonary embolism in the left lower lobe accompanied by mild inflammation and fibrosis, the diagnosis was confirmed. This process also has certain limitations. Although BALF morphology can serve as a core diagnostic tool and complement imaging to improve diagnostic accuracy, it is not 100% specific. Lipoid pneumonia, chronic aspiration, or other lipid-rich pulmonary diseases may also yield positive findings, necessitating strict correlation with clinical history and imaging findings. Furthermore, BALF is obtained via an invasive procedure, which limits its applicability in critically ill or hemodynamically unstable patients. In addition, sample quality, staining procedures, and morphological interpretation are operator-dependent, which may affect result consistency. As this is only a single case of delayed-onset pulmonary fat embolism following liposuction, the findings cannot be generalized to traumatic fat embolism, typical early-onset fat embolism syndrome, or other populations. Large-scale cohort studies are needed to validate the diagnostic value of BALF morphology.

Cases of pulmonary fat embolism caused by liposuction and fat grafting are widely reported. Nearly one-third of patients die, and over three-quarters require artificial airway placement and mechanical ventilation, indicating that pulmonary fat embolism is a devastating complication of liposuction and fat grafting (6). However, treatment and management strategies have not yet been fully established and remain primarily supportive, with a key aspect being the maintenance of oxygenation. A case report described a 29-year-old woman who developed fat embolism during liposuction and was successfully treated with a combination of mechanical ventilation, low-dose glucocorticoids, human albumin, and low-molecular-weight heparin, with no long-term complications. Nevertheless, in many traumatic cases, due to the lack of timely and accurate diagnosis and the extremely high associated mortality, fat embolism is often identified as the cause of death only at autopsy. This serves as a critical safety warning for aesthetic and plastic surgery. Although cosmetic surgery fulfills patients' desires for aesthetic enhancement, it is by no means a risk-free “minimally invasive procedure,” and its potential medical hazards should not be underestimated. Traditional diagnostic approaches rely on subjective methods and special staining techniques such as oil red O (7), which are inadequate to meet the clinical demand for rapid and accurate diagnosis. In this context, morphological examination of BALF highlights its irreplaceable importance. In the present case, BALF morphology not only complemented imaging findings such as chest CT but also allowed for efficient exclusion of other conditions with similar presentations, such as pneumonia and pulmonary edema, by directly visualizing lipid components in the lavage fluid, thereby securing a critical time window for early recognition and intervention. This case represents a delayed-onset mild pulmonary fat embolism following liposuction. The patient achieved symptom relief within 7 days after symptomatic treatment alone, with a relatively stable clinical course. However, in critically ill cases, BALF morphological examination can rapidly reveal key evidence, such as macrophages phagocytosing fat particles and free fat droplets, providing timely and direct diagnostic evidence for clinicians. This case highlights that after plastic surgery procedures such as liposuction, even beyond the typical 24–48 h onset window, the possibility of pulmonary fat embolism should be considered in patients presenting with symptoms such as chest tightness and dyspnea. BALF morphological examination holds irreplaceable value in the early recognition of critically ill patients, helping to save valuable time and avoid misdiagnosis or missed diagnosis. Plastic surgeons should prioritize risk assessment for all surgical procedures, establish long-term follow-up mechanisms, and promptly identify potential complications.

## Data Availability

The original contributions presented in the study are included in the article/supplementary material, further inquiries can be directed to the corresponding author.
